# Ecological and evolutionary perspectives on tick-borne pathogen co-infections

**DOI:** 10.1016/j.crpvbd.2021.100049

**Published:** 2021-09-25

**Authors:** Andrea Gomez-Chamorro, Adnan Hodžić, Kayla C. King, Alejandro Cabezas-Cruz

**Affiliations:** a Department of Zoology, University of Oxford, Oxford, OX1 3SZ, UK; b Anses, INRAE, Ecole Nationale Vétérinaire D’Alfort, UMR BIPAR, Laboratoire de Santé Animale, Maisons-Alfort, F-94700, France; c Institute of Parasitology, Department of Pathobiology, University of Veterinary Medicine Vienna, Veterinaerplatz 1, 1210, Vienna, Austria

**Keywords:** Tick-borne pathogens, Co-infections, Experimental evolution, Tick cell lines, Virulence, Transmission, Microbiota

## Abstract

Tick-borne pathogen co-infections are common in nature. Co-infecting pathogens interact with each other and the tick microbiome, which influences individual pathogen fitness, and ultimately shapes virulence, infectivity, and transmission. In this review, we discuss how tick-borne pathogens are an ideal framework to study the evolutionary dynamics of co-infections. We highlight the importance of inter-species and intra-species interactions in vector-borne pathogen ecology and evolution. We also propose experimental evolution in tick cell lines as a method to directly test the impact of co-infections on pathogen evolution. Experimental evolution can simulate in real-time the long periods of time involved in within-vector pathogen interactions in nature, a major practical obstacle to cracking the influence of co-infections on pathogen evolution and ecology.

## Highlights

• Co-infections are common in nature and influence morbidity, disease severity, and mortality in animals and humans.
• Multiple-pathogen co-infections affect colonization of the tick vector and transmission to the vertebrate host.
• Tick-borne pathogens hijack tick protein expression to modulate the microbiome and facilitate pathogen infection.
• Tick cell lines are a versatile platform to perform experimental evolution studies using multiple-pathogen infections.
• Experimental evolution and transmission experiments can elucidate the impact of co-infections on pathogen transmission.

## Graphical abstract

**Figure:**
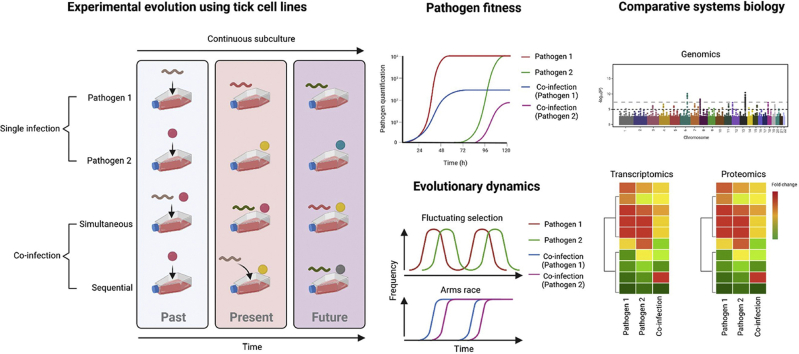

## 1 Introduction

Co-infections, whereby multiple pathogen species or genotypes coexisting within the same host, are very common in nature (Karvonen et al., 2019). A wide range of organisms, including humans (Balmer and Tanner, 2011), animals (Telfer et al., 2010), plants (Malpica et al., 2006), and bacteria (Turner and Duffy, 2008) can be hosts for multiple pathogens. The epidemiological and clinical implications of co-infections are widely recognized and considered a major veterinary and public health concern (Balmer and Tanner, 2011). Co-infections have impacts on disease severity with implications for diagnosis and treatment of infections (Balmer and Tanner, 2011; Johnson et al., 2015).

Co-infecting pathogens interact in ways that can be facilitative or competitive (Graham, 2008; Karvonen et al., 2019). Facilitative interactions occur when one species positively impacts the fitness of other species (Karvonen et al., 2012). Direct facilitation, such as *via* ‘supplied products’, can occur when substances produced by one species benefit other species. Facilitation can occur indirectly *via* host-mediated responses, when the suppression of the host immune system by one pathogen benefits another (Karvonen et al., 2012). Conversely, competitive interactions occur when each species negatively impacts the fitness of the other (Mideo, 2009). Pathogens can engage in exploitation competition for host resources (Gold et al., 2009) or interference competition, such as *via* toxin production (Bashey et al., 2013). Host-mediated competition can also occur indirectly *via* host responses. This is what happens when there is cross-immunity, and for example, when host antibodies produced to tackle one pathogen act against another pathogen (Cox, 2001).

Given the ability of a pathogen to grow and establish in a niche is impacted by co-infection, changes can occur in within-host pathogen fitness and transmission, and ultimately the evolution of virulence (Karvonen et al., 2012; Alizon et al., 2013; Susi et al., 2015). Pathogens themselves may also adapt to exist in co-infection (Mideo, 2009). The outcome of within-host interactions is often difficult to predict given the dependence on the species and strains involved, environment, mechanism, and timing (Seppälä and Jokela, 2016; Karvonen et al., 2019). For instance, a meta-analysis of helminth-microparasite co-infections found that resource limitation (e.g. competition for red blood cells) between the two taxa decreases the microparasite population size, whereas suppression of inflammatory immune responses has the opposite effect (Graham, 2008).

## 2 Ticks and tick-borne pathogens, a system to study co-infections

Arthropod vectors, such as ticks, provide an ideal framework to study co-infection in the context of pathogen evolution. First, co-infection is highly common in these vectors. Ticks are regularly found to be co-infected in the field (Michelet et al., 2014; Prusinski et al., 2014). Recent survey studies have found a higher co-infection prevalence (about 50% or more) than previously reported and up to five different pathogens were identified in single ticks (Michelet et al., 2014; Prusinski et al., 2014; Moutailler et al., 2016; Walter et al., 2016; Durand et al., 2017a, Durand et al., 2017b). Second, ticks accumulate multiple pathogen species and strains during their lifespan that can be co-transmitted to their vertebrate host (Levin and Fish, 2000). Third, ticks are among the most important pathogen vectors to domestic and wild animals, and, after mosquitoes, the most important vectors of pathogens affecting human health. They also vector multiple emerging diseases, such as Lyme disease, tick-borne encephalitis (TBE), and human granulocytic anaplasmosis (HGA) (de la Fuente et al., 2017). Fourth, ticks are laboratory-tractable and a number of cell lines have been shown to be effective for studying interactions between tick-borne pathogens *in vitro* (Bell-Sakyi et al., 2007; Moniuszko et al., 2014). Fifth, different state-of-the-art *omics* and high-throughput technologies can be applied to ticks and their pathogens to tease apart the mechanisms underlying changes in infection-related traits (Ayllón et al., 2015; Bekebrede et al., 2020; Lin et al., 2021). The use of this system can expand our knowledge on the ability of co-infections to shape the evolutionary biology of vector-borne pathogens.

Tick-pathogen interactions have been traditionally studied in the pairwise species model (i.e. one vector species infected by one pathogen species) (Cabezas-Cruz et al., 2018). Accordingly, the complexity of pathogen co-infection ecology within ticks remains to be thoroughly explored (Cutler et al., 2020). Ticks have long life-cycles of one to three years, with four developmental stages: egg, larva, nymph and adult (Bowman and Nuttall, 2008). Blood-feeding increases the possibility of acquiring pathogens that accumulate through the life-cycle. Of the 900 currently known tick species, at least 10% are reported to transmit pathogens of medical and veterinary importance (Bowman and Nuttall, 2008). Tick-borne pathogens include bacteria, viruses, protozoans and helminths. Over the past years, new tick-borne pathogens have been reported and this number is expected to keep growing (Cutler et al., 2020). Recently, the importance of tick-borne pathogen co-infections has become evident and is routinely considered in survey studies (Michelet et al., 2014; Moutailler et al., 2016). However, we should consider that co-detection of pathogens by PCR in field-collected ticks and their vertebrate host does not always indicate a viable co-infection. This may bias an overall picture of the ecology and evolution perspective of tick-borne pathogen co-infections. It is thus important to perform more experimental co-infection studies and establish new research models to overcome the issue.

## 3 Inter-species co-infections

Several pathogens transmitted by *Ixodes* species to their vertebrate hosts – such as spirochetal bacteria (e.g. *Borrelia burgdorferi* (*sensu lato*) and *Borrelia miyamotoi*), rickettsial bacteria (e.g. spotted fever group (SFG) rickettsiae and *Anaplasma phagocytophilum*), flaviviruses (e.g. tick-borne encephalitis virus, TBEV), and protozoan parasites (e.g. *Babesia microti*) – are highly prevalent in different regions of the world (de la Fuente et al., 2017). Some of the best characterized co-infections are those caused by pathogens transmitted by *Ixodes scapularis* in the USA: *B. burgdorferi* + *A. phagocytophilum* and *B. burgdorferi* + *B. microti*. These three pathogen species are of clinical importance as the etiological agents of Lyme disease (*B. burgdorferi*), human granulocytic anaplasmosis (*A. phagocytophilum*), and human babesiosis (*B. microti*).

The association between *B. burgdorferi* (*s.l.*) and *A. phagocytophilum* is one of the best studied examples of tick-borne pathogen co-infection. These two species have been frequently reported to occur together in ticks, wild animals, and some clinical cases of human infections (Nieto and Foley, 2009; Bakken and Dumler, 2015). They were found to co-occur in 3–15% of patients with a tick-borne infection in some regions of the USA (Belongia, 2002). Their co-infection can increase morbidity, bacterial load, and severity of symptoms (Holden et al., 2005; Thomas et al., 2001). A significant increase of Lyme arthritis was reported in mice experimentally co-infected with *B. burgdorferi* (*s.l.*) and *A. phagocytophilum* (Thomas et al., 2001), and *A. phagocytophilum*-infected neutrophils enhance the transmigration of *B. burgdorferi* across the human blood-brain barrier (Nyarko et al., 2006). Co-infections also elicit different immune responses within mice hosts. For example, the antibody response to *A. phagocytophilum* decreased during co-infection, but antibodies produced in response to *B. burgdorferi* increased in co-infected mice (Holden et al., 2005).

**Fig.1:**
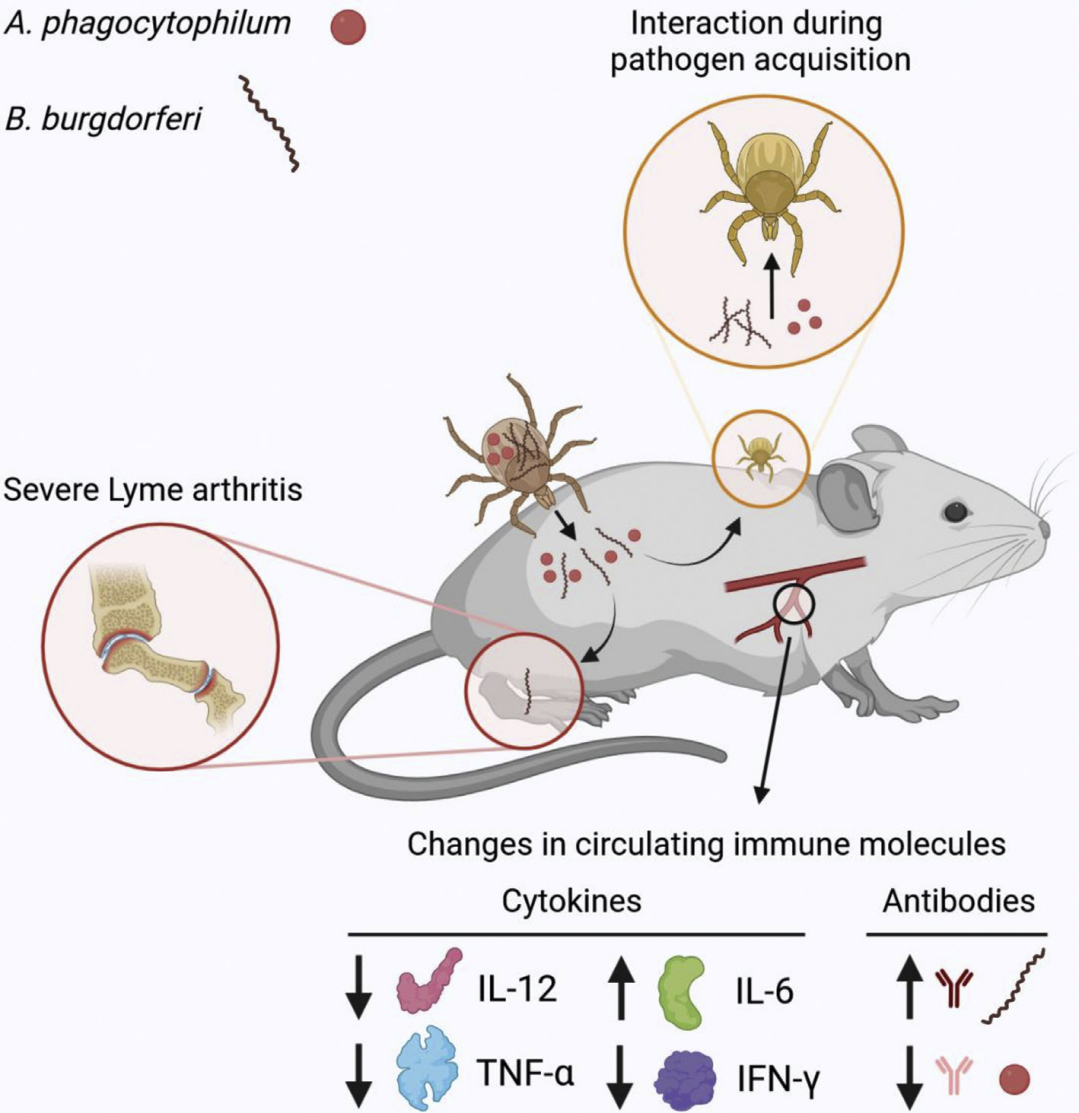
Interactions between co-infecting *A. phagocytophilum* and *B. burgdorferi*. Simultaneous or sequential transmission of *A. phagocytophilum* and *B. burgdorferi* could result in co-infection. Mice infected with these two bacteria show a more severe Lyme arthritis (Thomas et al., 2001), an inflammatory pathology caused by the entrance of *B. burgdorferi* in the joint tissue. Co-infected mice also show changes in the levels of inflammatory cytokines (i.e. IL-12, IL-12, TNF-α, and IFN-γ) (Thomas et al., 2001). The antibody response to *B. burgdorferi* and *A. phagocytophilum* increased and decreased, respectively, in co-infected animals (Holden et al., 2005). Individual pathogen acquisition by tick larvae have also been reported to be affected by this co-infection (Levin and Fish, 2000; Thomas et al., 2001).

Tick infection and colonization by *A. phagocytophilum* and *B. burgdorferi* occurs firstly in the gut cells and subsequently in other tissues, including the salivary glands from where transmission occurs during feeding. Thus, these pathogens coexist and potentially interact within the same tissues for long periods of time. Evidence shows that the interactions between *A. phagocytophilum* and *B. burgdorferi* are not neutral (Fig. 1). However, whether these bacteria facilitate their mutual infection or compete for common ecological niches remains controversial. Some experimental co-infection studies showed that the presence of these two pathogens in the animal host enhances acquisition of both bacteria by tick larvae (Thomas et al., 2001), while other report suggested interference between these two agents during the transfer from co-infected mice to larvae (Levin and Fish, 2000).

*Borreliaburgdorferi* (*s.l.*) complex comprises 21 recognized genospecies, and ticks can be simultaneously infected with more than one *Borrelia* species (Hovius et al., 2007; Michelet et al., 2014). Additionally, more than 50% of Lyme disease patients reported a co-infection with at least an additional tick-borne pathogen (Johnson et al., 2014). Empirical evidence shows that the presence of two pathogen species (i.e. *B. burgdorferi* (*sensu stricto*) and *Borrelia garinii*) altered murine Lyme borreliosis by enhancing pathogen burden and resulted in a more severe disease outcome (Hovius et al., 2007). This led the authors to suggest that co-infection could lead to preferential maintenance and a rising prevalence of *B. burgdorferi* (*s.s.*) in European ticks (Hovius et al., 2007). In contrast, some genospecies rarely co-infect the same tick due to their adaptation to different reservoir hosts, such as *Borrelia afzelii* and *B. garinii*, commonly associated with rodents and birds, respectively (Kurtenbach et al., 2001; Herrmann et al., 2013).

Emerging and re-emerging tick-borne pathogens have been described and characterized in endemic settings. One interesting case is the co-infection with *B. burgdorferi* and *B. microti* (Diuk-Wasser et al., 2016). In some regions of the USA, the proportion of *Ixodes* ticks co-infected with *B. burgdorferi* and *B. microti* is higher than that of *A. phagocytophilum* + *B. burgdorferi* co-infection (Belongia, 2002). Notably, in recent years, the range and prevalence of *B. microti* has increased significantly in the northeastern USA where *B. burgdorferi* has been historically highly prevalent, suggesting important epidemiological consequences of this interaction (Diuk-Wasser et al., 2016). An experimental study demonstrated that *B. burgdorferi* (*s.s*.) promotes the establishment of *B. microti* in *Peromyscus leucopus* mice and that larval ticks are infected with *B. microti* in higher numbers when fed on mice co-infected with *Borrelia* spirochetes (Dunn et al., 2014). *B. burgdorferi* + *B. microti* co-infection may create an immunological conflict that increases *B. microti* fitness, higher parasitemia, and transmission rate to feeding ticks (Diuk-Wasser et al., 2016). Other infections such as bartonellosis, caused by *Bartonella* spp., have been recognized as an emerging or re-emerging zoonosis and found in co-infection with *B. burgdorferi*. The role of *Bartonella* spp. as a tick-borne pathogen is nevertheless under discussion (Maggi et al., 2019).

Little is known about the impact of co-infections in ticks on the transmission of individual pathogens to humans. Recently, using a pairwise sampling approach, *Ixodes ricinus* ticks feeding on human and blood samples from the same individuals were screened by a microfluidic real-time high-throughput PCR system detecting several tick-borne microorganisms (Banović et al., 2021). Surprisingly, despite a high infection rate of single infection (74%) and co-infections (38%) in ticks, only two human blood samples tested positive for the presence of tick-borne pathogens. One patient was diagnosed with *Borrelia* spp. and the other was diagnosed with *Rickettsia felis* infection. The tick infesting one of the patients tested positive for *B. afzelii*, and *Rickettsia helvetica*, while the other tick tested positive only for *R. felis* (Banović et al., 2021). However, the absence of the pathogens in the blood of the tested patients does not necessarily mean that those individuals were not infected. These results warrant further research to decipher whether pathogen interference or enhancement occurs within the vector causing an altered probability of single pathogen transmission to humans. Another study explored the association between the genetic diversity of *Ehrlichia canis* and co-infections in *Rhipicephalus sanguineus* ticks on dogs (Cabezas-Cruz et al., 2019a). *Rickettsia massiliae* and *E. canis* were the most common co-infecting pathogens. Strain analysis allowed the identification of three *E. canis* strains with low genetic diversity, and one of the strains appeared to be more adapted to co-infection with *R. massiliae* (Cabezas-Cruz et al., 2019a).

## 4 Co-infections with strains of the same pathogen species

Additional layers of complexity are introduced to the multi-pathogen system by intra-species interactions (Kurtenbach et al., 2006). Theory predicts that interactions between closely related pathogens (strains of the same species) should be stronger because of similarities in the transmission routes and/or use of resources (Alizon et al., 2013). Pathogen strains can vary in their infectivity, transmission, and virulence (Karvonen et al., 2012; Alizon et al., 2013; Susi et al., 2015). Therefore, the strain composition can influence the epidemiology and evolution of these pathogens. Co-infections by multiple strains of the same tick-borne pathogen species are common in tick vector and vertebrate hosts (Palmer et al., 2004; Castañeda-Ortiz et al., 2015; Hove et al., 2018). Some of the most studied examples are co-infections with *B. burgdorferi* (*s.l.*), *A. phagocytophilum* and *Anaplasma marginale* (Castañeda-Ortiz et al., 2015; Walter et al., 2016; Langenwalder et al., 2020), but co-infections also occur between strains of other tick-borne pathogens that are less commonly screened.

Field studies using deep sequencing found that 70–80% of ticks can be co-infected with multiple strains of *Borrelia* and positive and negative interactions between strains of *Borrelia* species were detected in ticks (Walter et al., 2016; Durand et al., 2017a, Durand et al., 2017b). In addition, competition experiments between *B. afzelii* strains showed that strain interaction affects bacterial density and prevalence in immature *I. ricinus* ticks (Genné et al., 2018). Considering that *B. burgdorferi* density within ticks positively correlates with the probability of transmission to vertebrate hosts (Rego et al., 2014), it is expected that competition among co-infecting strains may reduce the evolutionary fitness of the subdominant strain (Oppler et al., 2021). Competition among co-infecting strains could select for traits to suppress the growth of other strains or to escape suppression. However, there is no evidence that such traits have evolved in *Borrelia* and the evolutionary pressures that may produce them are currently unknown (Oppler et al., 2021). Future research could investigate experimentally the selective pressures created by competition between co-infecting *Borrelia* strains and determine their relative evolutionary outcomes.

High strain diversity has also been reported for the tick-borne pathogens *A. phagocytophilum* and *A. marginale* (Palmer et al., 2004; Castañeda-Ortiz et al., 2015; Hove et al., 2018). Considerable strain variations of *A. phagocytophilum* have been reported and several studies demonstrated the correlation of the bacterial genotypes and the vertebrate hosts, suggesting that host preference is an important contributor to strain diversity in this pathogen (Scharf et al., 2011; Huhn et al., 2014). High numbers of *A. phagocytophilum* haplotypes have been recorded in the tick *I. ricinus* (Jaarsma et al., 2019), which is in agreement with an earlier study showing that 41% of *I. ricinus* can be infected with more than one strain of *A. phagocytophilum* (Huhn et al., 2014). Circulation of different strains in a cattle herd over one pasture season has also been demonstrated in a recent comprehensive molecular study in Germany (Langenwalder et al., 2020). Whether inter-strain competition exists for this pathogen within the vector or whether ticks carrying multiple *A. phagocytophilum* strains can transmit them simultaneously to cattle or other hosts remains an open question. However, genetically distinct strains of the closely related pathogen *A. marginale* are capable of being co-transmitted by ticks and superinfecting cattle (Leverich et al., 2008). Co-infections by multiple *A. marginale* strains have been detected in unvaccinated cattle populations where this bacterium is endemic (Palmer et al., 2004). Interestingly, superinfection with different *A. marginale* strains was also reported without a predominance of any of the strains in the herd for a period of five years, which was explained by the occurrence of preferential strain transmission within a population due to stochastic pathogen transmission (Palmer et al., 2004). In contrast to co-infecting *B. burgdorferi* strains in which low density was associated with low transmission probability (Rego et al., 2014), successful transmission of co-infecting *A. marginale* strains was independent of the bacteremia levels (Leverich et al., 2008).

## 5 Co-infections and the tick microbiome

Tick-borne pathogens coexist and interact with several bacterial species of the tick microbiome. The ticks and their associated microbial communities can form an ecological unit, called the tick holobiont (Díaz-Sánchez et al., 2019). The contributions of the tick microbiota to tick-pathogen interactions are highly relevant for vectorial capacity, with the relationship between microbiota and pathogens being bidirectional (Wu-Chuang et al., 2021a). For example, tick colonization by *A. phagocytophilum* or *B. afzelii* has been reported to modulate the tick microbiome (Abraham et al., 2017; Hamilton et al., 2021). Ticks bred in a sterile environment without microbiota have altered gut integrity which reduced the ability of *B. burgdorferi* to colonize this niche (Narasimhan et al., 2014). In the case of *B. burgdorferi*, the normal tick microbiota can facilitate pathogen infection as reported in other host-pathogen systems (Stevens et al., 2021).

Empirical work suggests that *A. phagocytophilum* and *B. burgdorferi* interactions can be mediated by the tick vector and its microbiome, with a single infection disrupting the resting state of tick-microbiome homeostasis. *Anaplasma* *phagocytophilum* induces ticks to express an anti-freeze glycoprotein (IAFGP) with the ability to alter bacterial biofilm formation and tick microbiota composition (Abraham et al., 2017). IAFGP-dependent modulation of tick microbiota influences the integrity of the peritrophic matrix (PM) and gut barrier, which are obstacles for *A. phagocytophilum* colonization (Abraham et al., 2017), but protects *B. burgdorferi* from toxic components of the gut lumen (Narasimhan et al., 2014). Accordingly, *B. burgdorferi* colonization increases the expression of *pixr*, a tick gene encoding a protein with a Reeler domain involved in the maintenance of the PM integrity and associated with the inhibition of bacterial biofilm formation (Narasimhan et al., 2014). Both IAFGP and PIXR are tick molecules hijacked by these tick-borne pathogens to regulate the tick microbiome homeostasis.

The nature of the relationship between pathogen co-infection and vector microbiome composition remains unclear. The association between microbiome composition and co-infections in ticks submitted for diagnostic testing was recently assessed (Gil et al., 2020). The microbiome of whole *I. scapularis* nymphs and adults that tested positive for one, two or three tick-borne pathogens (i.e. *B. burgdorferi*, *B. miyamotoi*, *A. phagocytophilum* and *B. microti*) was compared with that of uninfected ticks. In the study by Gil et al. (2020) no significant differences were found in the alpha- and beta-diversity indices of the tick microbiome under single or co-infections with any of the pathogens tested (Gil et al., 2020). However, other studies (e.g. see Sperling et al., 2020) found that *B. burgdorferi* infection does alter the tick microbiome. Particularly, ticks that were qPCR-positive for *Borrelia* had significantly greater bacterial diversity than *Borrelia*-negative ticks (Sperling et al., 2020). Further empirical studies are needed to directly test hypotheses on the relationship between co-infections and the tick microbiome.

**Fig. 2:**
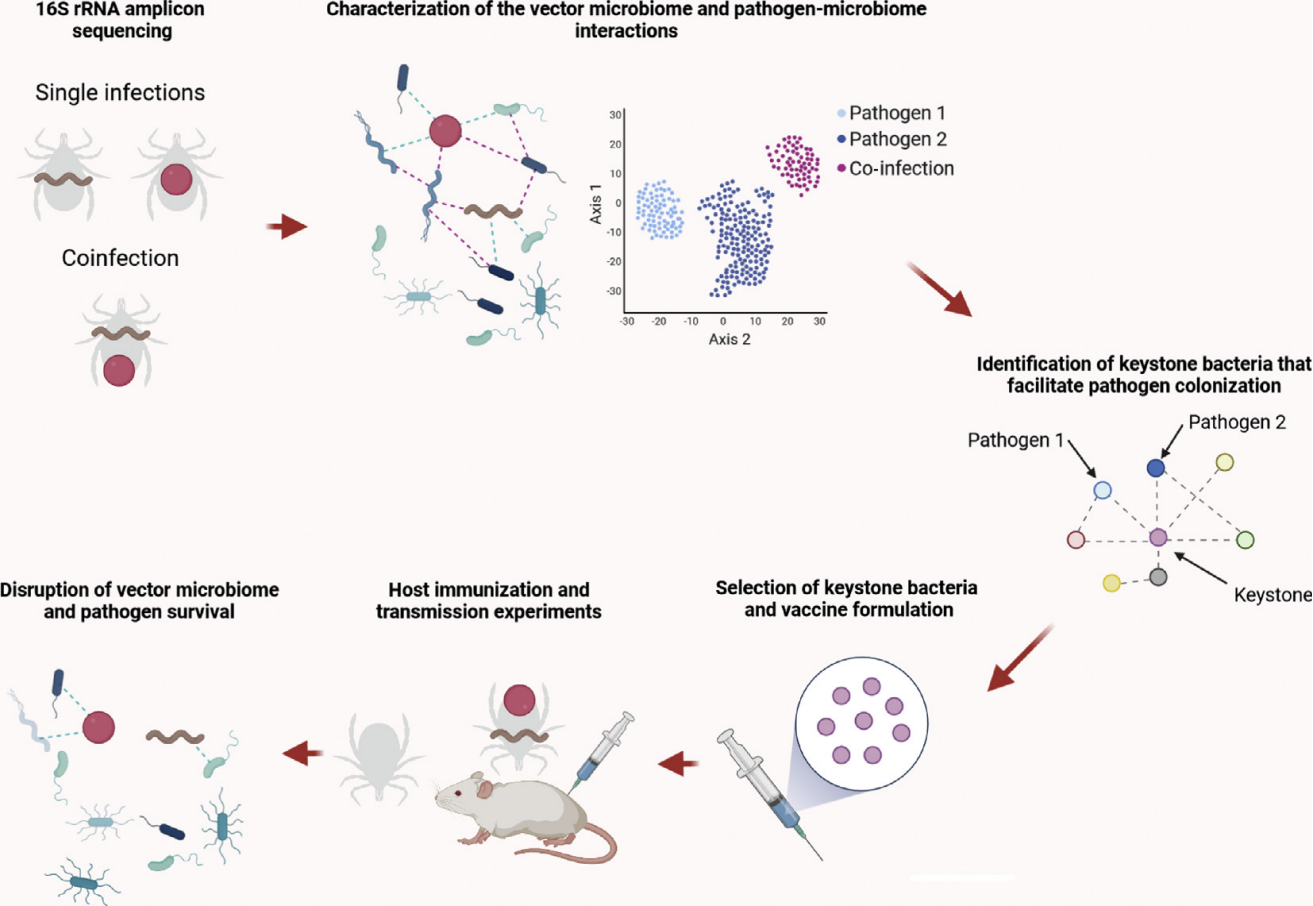
Disrupting vector-pathogen-microbiome interactions with anti-microbiota vaccines. Using 16S rRNA amplicon sequencing we can characterize the taxonomic profiles of the tick microbiome under single and co-infections. Co-occurrence networks can be used to identify keystone bacteria potentially involved in facilitation of individual pathogens or multi-pathogen infection. Selected keystone bacteria can be used in anti-microbiota vaccines, formulated as previously described (Mateos-Hernández et al., 2020, 2021a), to induce bacteria-specific antibodies in mice. Disruption of the tick microbiome with antibodies targeting the keystone bacteria could potentially block pathogen colonization and transmission.

Alterations of tick microbiomes may be a fruitful avenue for disrupting pathogen transmission (Shaw and Catteruccia, 2019). Progress in molecular and mechanistic insights into the tick microbiome has nevertheless been hindered by technical difficulties in manipulating the microbiome. Recent advances, however, show that anti-microbiota vaccines are a suitable tool to manipulate the tick microbiome in a taxon-specific manner (Mateos-Hernández et al., 2020, 2021a; Wu-Chuang et al., 2021b). Immunization of mice with an *Escherichia coli* live vaccine targeting a keystone genus of the tick microbiome, *Escherichia*-*Shigella*, reduced microbiota diversity in *I. ricinus* (Mateos-Hernández et al., 2021a) and a significant negative correlation between *Escherichia*-*Shigella* abundance and the levels of host antibodies (i.e. IgM and IgG) specific to *E. coli* proteins suggested that the effect of the anti-microbiota vaccine is taxon-specific and mediated by host antibodies (Mateos-Hernández et al., 2020, 2021a). Immunization against the keystone bacteria restructured the hierarchy of the microbial community in ticks and decreased the keystoneness of *Escherichia*-*Shigella* in the co-occurrence networks (Mateos-Hernández et al., 2021a). These results opened up the possibility of using anti-microbiota vaccines as a tool for experimental manipulation of the tick microbiome and potentially block tick-borne pathogen transmission (Fig. 2).

## 6 Tick cells as a tool to measure evolutionary interactions between tick-borne pathogens

Tick cell lines constitute a useful model to study tick-pathogen interactions (Bell-Sakyi et al., 2007; Cabezas-Cruz et al., 2019b) and co-infections (Moniuszko et al., 2014). Experimental evolution approaches can be applied to this model because tick cells are handy, easy to manipulate, have a good growing pace, and capable of being stored short-term at 4 °C. Many tick cell lines derived from embryonic cells of different tick species are available at the Tick Cell Biobank (The Pirbright Institute, UK) (Bell-Sakyi et al., 2007). *In vitro* culture of major tick-borne pathogens such as *A. phagocytophilum* (Munderloh et al., 1999), *A. marginale* (Passos, 2012) *Ehrlichia chaffeensis* (Singu et al., 2006), *E. canis* (Singu et al., 2006; Ferrolho et al., 2016), *Ehrlichia ruminantium* (Moniuszko et al., 2014), *B. burgdorferi* (Obonyo et al., 1999; Bugrysheva et al., 2002), and TBEV (Weisheit et al., 2015) have been established in these cells. Several tick-borne pathogens can be propagated in the same tick cell line, which provides the opportunity to model multi-pathogen infection systems within the same tick vector cells. For example, *E. ruminantium* (Ferrolho et al., 2016), a North American strain of *E. canis* (Singu et al., 2006), and European strains of *B. burgdorferi* (Obonyo et al., 1999) and *A. phagocytophilum* (Munderloh et al., 1999) were grown *in vitro* in ISE6 cells. Another advantage is that single tick-borne pathogens can be propagated in different tick cell lines. For example, efficient replication of TBEV was achieved in both *I. scapularis*-derived cell line IDE8 and the *I. ricinus*-derived cell line IRE/CTVM19 (Weisheit et al., 2015). Notably, there is one study of tick-borne pathogens co-infection using ISE6 and IRE/CTVM19 tick cell lines (Moniuszko et al., 2014). In their pioneering work, Moniuszko et al. (2014) showed an asymmetrical interaction between *B. burgdorferi*, *E. ruminantium*, or Semliki Forest virus (SFV) in tick cell culture. The presence of *B. burgdorferi* had a positive effect, enhancing the replication of *E. ruminantium* and SFV, but no other interaction showed any difference.

Importantly, studies using tick cell lines have also found complex biological responses in pathogens and vector cells that indicated specific tick cell-pathogen interactions (Bugrysheva et al., 2002; Cabezas-Cruz et al., 2019b). These interactions have been described in both intracellular (e.g. *A. phagocytophilum* and TBEV) and extracellular (i.e. *B. burgdorferi*) tick-borne pathogens. While interactions between tick cells and intracellular pathogens are expected and have been characterized *in vitro* in some tick-borne pathogens such as *A. phagocytophilum* (Cabezas-Cruz et al., 2017a, b, 2019b) and TBEV (Weisheit et al., 2015), studies of tick cell-*B. burgdorferi* interactions *in vitro* are comparatively less represented in the literature. Several lines of evidence, however, show reciprocal interactions between *B. burgdorferi* and tick cells *in vitro*. First, *B. burgdorferi* replicates in L15BS when co-cultured with tick cell lines, while the spirochete did not grow in L15BS medium alone (Bugrysheva et al., 2002). Second, *B. burgdorferi* adhered tightly to tick cells (Bugrysheva et al., 2002). Third, coculture of *B. burgdorferi* with tick cells modulates bacterial gene expression (Obonyo et al., 1999; Bugrysheva et al., 2002). In tick cell culture, *B. burgdorferi* modulated the expression of outer surface proteins A and C in response to temperature changes (Obonyo et al., 1999), decreased the mRNA levels of *relA*/*spot* and *bmpD*, and increased *rpsL*-*bmpD* levels (Bugrysheva et al., 2002). Altogether, this suggests tick cell lines are a relevant model to study interactions in both intracellular and extracellular pathogens.

The mechanisms underlying pathogen interactions can be studied in tick cells using state-of-the-art transcriptomics, metabolomics and proteomics approaches as well as gene-silencing techniques such as RNA interference (Ayllón et al., 2015; Weisheit et al., 2015; Villar et al., 2015; Cabezas-Cruz et al., 2017a,b). The transcriptional and protein response of tick cells to pathogen infection (i.e. *A. phagocytophilum*) can partially mirror that of tick tissues such as midgut and salivary glands (Cabezas-Cruz et al., 2017a, b). In fact, some functional and morphological features of tick tissues have been described in tick cell lines (Oliver et al., 2015; Mateos-Hernández et al., 2021b). Changes in the abundance of several enzymes involved in carbohydrate metabolism (e.g. hexokinase, fructose-bisphosphate aldolase, and pyruvate dehydrogenase E1) were similar in tick salivary glands and ISE6 cells upon *A. phagocytophilum* infection (Cabezas-Cruz et al., 2017b).

**Fig. 3:**
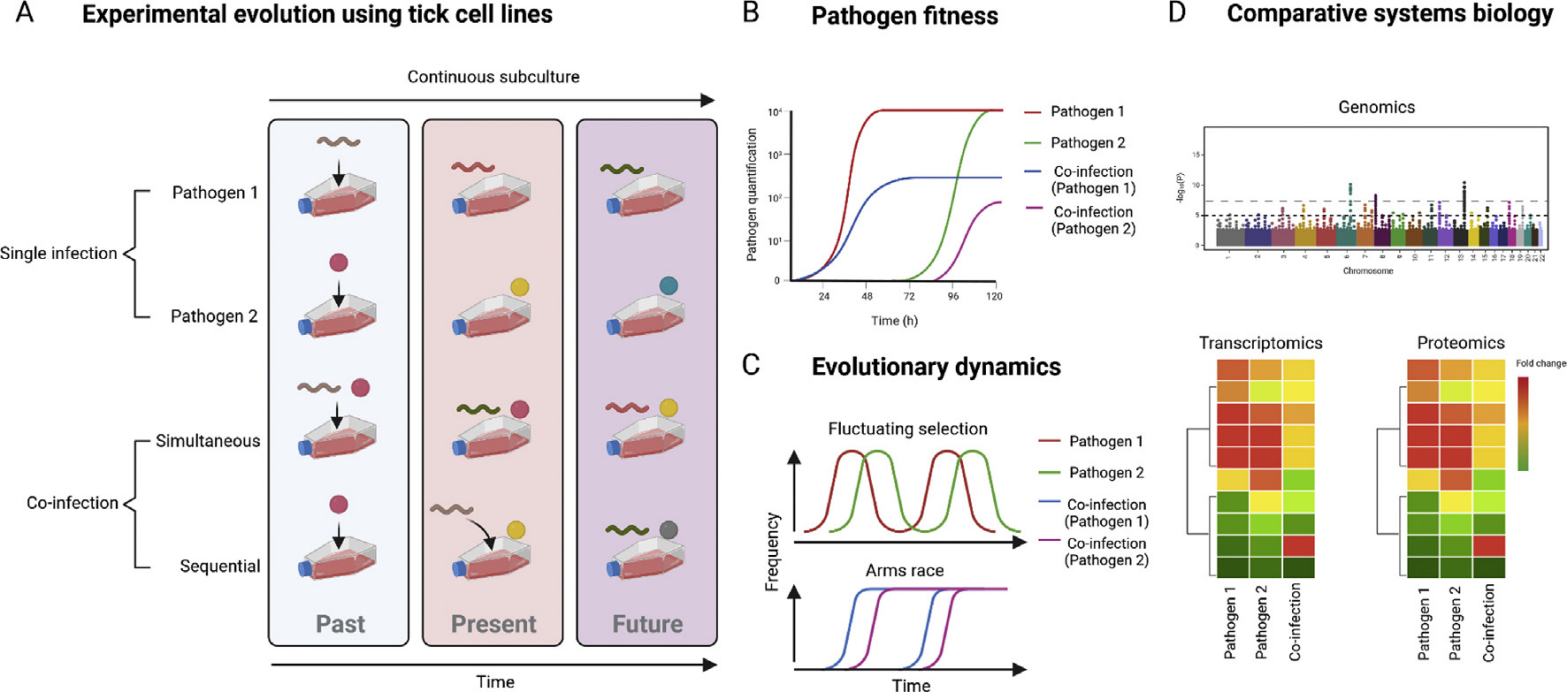
Experimental evolution in tick cells. **A** Tick cells offer a versatile system in which selected pathogens could be cultured under single and different modes of co-infections (i.e. simultaneous or sequential). The same tick cell line can be infected with several pathogens which can be continuously subcultured to assess short-term (e.g. 3–5 passages) and/or long-term evolution (e.g. 10–15 passages). **B** Pathogen fitness under single infections and co-infections can be measured by real time PCR and compared between groups and modes of evolution or coevolution (i.e. fluctuating selection and arms race) can be assessed (**C**). **D** Comparative systems biology approaches such as genomics, transcriptomics, and proteomics can be used following bouts of evolution to measure genetic and phenotypic changes of pathogens during co-infections. The same approaches (e.g. transcriptomics and proteomics) can be used to test the response of tick cell to multi-pathogen infection.

Cells further offer the possibility to use controlled loads of individual pathogens in co-infection studies. Experimental manipulations can be conducted either simultaneously or sequentially, whereby one pathogen is inoculated after the other with a time difference. All of these empirical and biological features make tick cells a versatile and flexible experimental system to simulate different evolutionary scenarios (Fig. 3A) using experimental evolution approaches. Pathogen fitness (Fig. 3B) and evolutionary dynamics (Fig. 3C) can be assessed following passage under single- and co-infections. An additional advantage of tick cells is the possibility of using comparative systems biology to elucidate genetic and phenotypic changes (Fig. 3D) associated with co-infections in vector cells, following experimental passage of pathogens.

**Fig. 4:**
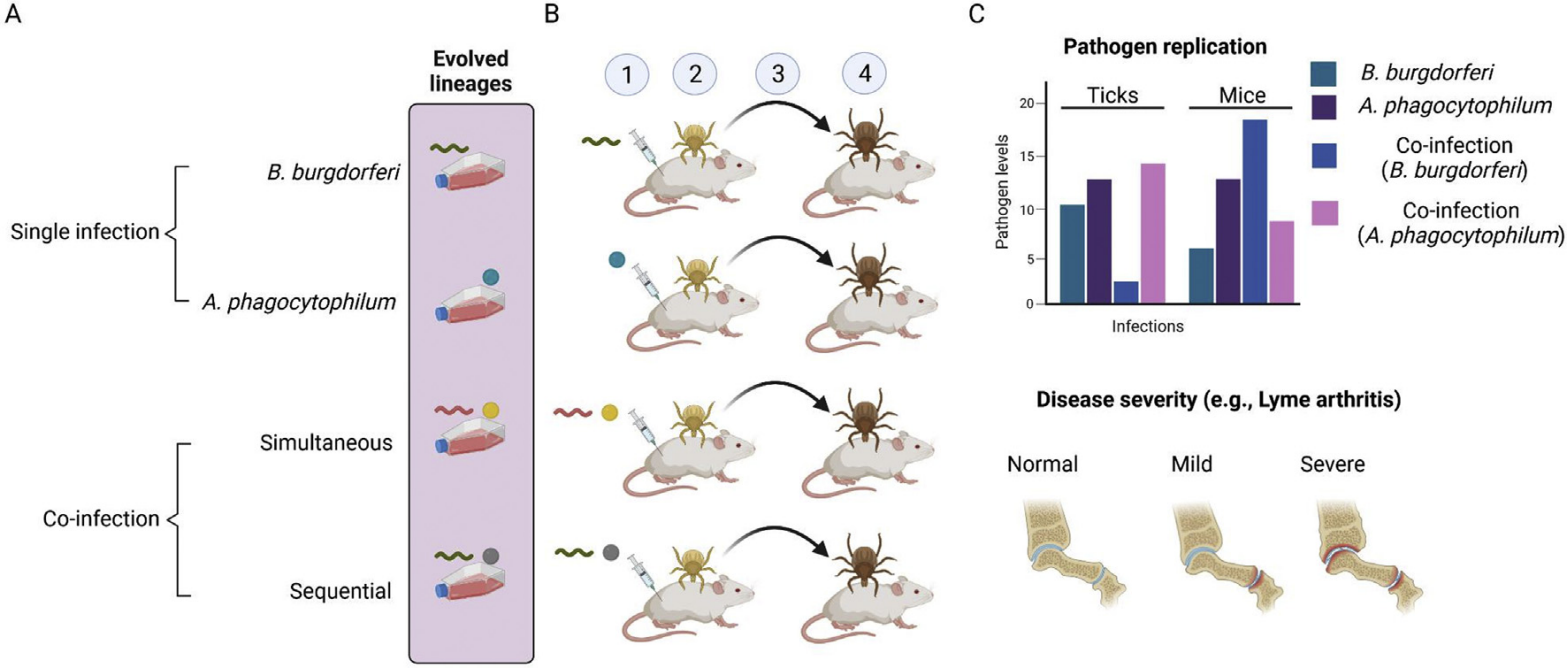
Impact of co-infections and potential coevolution on infection-related traits of *B. burgdorferi* and *A. phagocytophilum*. **A** Lineages of *B. burgdorferi* and *A. phagocytophilum* experimentally coevolved under single infection and co-infection under short-term and/or long-term continuous subculture can be used in transmission experiments. **B** Evolved pathogens can be inoculated in susceptible mice host (1), followed by tick larvae infestation for pathogen acquisition (2). After molting (3), infected nymphs are placed on naïve mice for pathogen transmission (3). **C** Pathogen replication in ticks and mice can be measured by real time PCR and compared between groups. The impact of co-infection on murine Lyme borreliosis severity (e.g. Lyme arthritis) can also be measured.

Although co-infections are pervasive in nature, experimental analysis of the impact of co-infection on vector-borne pathogen evolution and transmission has yet to be undertaken. Recent advances in *in vitro* culture of tick-borne pathogens within tick cell lines could allow testing whether co-infection affects pathogen evolution under experimental evolution and whether evolved pathogens experience changes in their virulence, infectivity, and transmissibility from ticks. Experimental evolution is a powerful tool to test the adaptation of parasites under certain conditions (Ebert, 1998), including during co-infections *in vivo* (Ford et al., 2016, Ford et al., 2017). We can reconcile *in vitro* experimental evolution, systems biology, and *in vivo* experiments to understand how co-infections impact the evolution and transmission of vector-borne pathogens and disease severity. For example, using lineages of *B. burgdorferi* and *A. phagocytophilum* experimentally evolved under single and co-infection (Fig. 4A), we could test the impact of co-infection on pathogen colonization in ticks and transmission to the vertebrate hosts (Fig. 4B), as well as disease severity (Fig. 4C). If the fitness of either *B. burgdorferi* or *A. phagocytophilum* decreases during co-infection, we expect that tick tissue colonization by one pathogen will hamper the other.

Once ‘archived’ (i.e. not evolved), selected lineages can be selected and paired such that fitness can be assessed with past or future generations (i.e. time shift assays). These time-shift assays can elucidate some modes of selection during coevolution, should evolution be reciprocal. In time-shift experiments, samples of host (or pathogen) populations from different time points are tested in combination with samples of pathogen (or host) populations from other moments in time (Gaba and Ebert, 2009). In time-shift experiments, the mode of selection underlying coevolution can be revealed by measuring the fitness of one pathogen population exposed to the past and future population of the second pathogen population (Gaba and Ebert, 2009). Time-shifts are powerful tests that allow differentiating between modes of coevolution: arms-race dynamics and fluctuating selection dynamics (Agrawal and Lively, 2001). In arms race dynamics, one species adapts and reduces the fitness of individuals in a second species, which favours the selection of counter-adaptations in the second species. These counter-adaptations will also select in favour of new adaptations in the first species. A monotonous decrease of bacteria fitness from the past to the present to the future is considered a signature of an escalating coevolutionary arms race. In fluctuating selection dynamics, pathogen fitness is highest against antagonists from the recent past but less infective against antagonists from further in the past (Agrawal and Lively, 2001). Time-shift assays have revealed evidence of fluctuating selection dynamics in other co-infecting systems (Ford et al., 2017).

Evolutionary approaches will advance the state-of-the-art by directly testing the contribution of co-infection to pathogen evolution and transmission. Adding an evolutionary perspective to co-infections is important because, as shown for other pathogen models it can reveal new virulence attributes and mechanisms by selecting for adaptive mutations (Stern et al., 2017; Graf et al., 2021). Moreover, experimental evolution can help understand the emergence of strains with changes in important pathogen traits (e.g. virulence and transmissibility), critical for outbreak responses and for the design of control measures in endemic areas. Notably, previous work in *B. afzelii* showed that the strain-specific estimates of reproduction number (R_0_) in laboratory mice explained over 70% of the variation in the prevalence of the strains in local tick populations (Durand et al., 2017a, Durand et al., 2017b). The strain-specific estimates of R_0_ were calculated using three important fitness components of tick-borne pathogens such as host-to-vector transmission, vector-to-host transmission, and co-feeding transmission. Results from experimental evolution could be used to predict variation in the transmission frequency of vector-borne pathogens under co-infections in different regions. Nonetheless, small population sizes, limited time-scales, and the simplified nature of lab environments may limit the generalizability of outcomes of experimental evolution (Kawecki et al., 2012).

## 7 Conclusions and perspectives

The continuous exploitation of environmental resources and the increase in human outdoor activities, has enhanced exposure to tick bites. The emergence and resurgence of tick-borne pathogens have followed. Ticks provide an ideal framework in which to study the impact of co-infection on pathogen evolution and transmission because they are frequently co-infected, accumulate and transmit multiple pathogen groups (i.e. bacteria, viruses, protozoans, and helminths) and strains. Recent advances in tick microbiome manipulation with anti-microbiota vaccines will help elucidate the role of the tick microbiome in single and co-infections. Despite recent advances in the study of inter-species and intra-species co-infections, most tick-pathogen-microbiome molecular interactions have been characterized in ‘single tick-pathogenʼ systems, major questions remain unanswered. The laboratory-tractable nature and availability of several tick cell lines for *in vitro* experimentation permit direct tests of the evolutionary processes and outcomes of tick co-infections. Such evolution experiments have been valuable in uncovering the role of competition in free-living microbial systems (Rainey and Travisano, 1998), and in some host environments (Ford et al., 2016, Ford et al., 2017) with relevance to pathogen virulence (Mackinnon and Read, 2004). Their utility in illuminating the impact of co-infection in vector-borne pathogen evolution and transmission remains to be tackled.

## Funding

No specific funding was received for this work.

## CRediT author statement

Andrea Gomez-Chamorro: Writing - Original Draft, Writing - Review & Editing. Adnan Hodžić: Writing - Review & Editing. Kayla C. King: Conceptualization, Writing - Original Draft, Supervision. Alejandro Cabezas-Cruz: Conceptualization, Visualization, Writing - Original Draft, Supervision.

## Declaration of competing interests

The authors declare that they have no known competing financial interests or personal relationships that could have appeared to influence the work reported in this paper.
